# P39 United Nations Sustainable Development Goals (SDGs): fighting antimicrobial resistance (AMR) by innovative antibiotic dashboard

**DOI:** 10.1093/jacamr/dlaf118.046

**Published:** 2025-07-14

**Authors:** Rasha Abdelsalam-Elshenawy, Nkiruka Umaru, Zoe Aslanpour

**Affiliations:** School of Health, Medicine and Life Sciences, University of Hertfordshire, AL10 9AB, UK; School of Health, Medicine and Life Sciences, University of Hertfordshire, AL10 9AB, UK; School of Health, Medicine and Life Sciences, University of Hertfordshire, AL10 9AB, UK

## Abstract

**Background:**

Antimicrobial resistance (AMR) threatens global health and progress towards the United Nations Sustainable Development Goals (SDGs), particularly SDG 3.^1^ Strengthening antimicrobial stewardship (AMS), using frameworks like ‘Start Smart, Then Focus’, is essential.^2^ The COVID-19 pandemic has intensified the AMR crisis, highlighting the urgent need for innovative tools like an antibiotic dashboard.^3,4^

**Objectives:**

To explore antimicrobial stewardship and propose an innovative antibiotic dashboard.

**Methods:**

This research consisted of three sequential studies. Study one conducted a systematic literature review on AMS implementation in acute care settings prior to and during the COVID-19 pandemic. Study two was a retrospective cross-sectional review of 640 medical records, evaluating antibiotic prescribing in hospitalized adults with respiratory infections. Study three involved a prospective survey of 240 healthcare professionals, exploring their knowledge, attitudes, and perceptions towards antibiotic prescribing and AMS. Ethical approval was secured, and public and patient involvement through the Citizens Senate was integral, with registration in ISRCTN.

**Results:**

Study One identified key antimicrobial stewardship (AMS) strategies in acute care settings, with prospective audits (85%) and quality metrics (77%) being the most prevalent. Study Two demonstrated effective AMS interventions, achieving antibiotic discontinuation in 47% of cases and de-escalation to narrow-spectrum antibiotics in 37%. Study Three highlighted the COVID-19 pandemic’s disruptive impact, with interruptions in antibiotic reviews (81%), AMS audits (70%), and IV-to-oral switches (67%). Based on these findings, an Antimicrobial Stewardship Dashboard was developed to visualize trends in antibiotic prescribing, bacterial infections, AMS interventions, and hospital outcomes, supporting data-driven improvements in AMS practices (Figure 1).

**Conclusions:**

This research highlights the urgent need to strengthen AMS to combat the escalating threat of antimicrobial resistance, supporting the objectives of the United Nations SDGs. The innovative Antibiotic Dashboard introduced in this study provides essential AMS metrics that enhance antibiotic management and improve patient outcomes. By featuring dynamic sections for both initial empirical therapy and pathogen-directed therapy, the dashboard facilitates real-time clinical decision-making, which is especially critical during health emergencies such as the COVID-19 pandemic. Implementing such a digital innovation is vital to sustaining effective AMS practices, expanding research opportunities, and advancing global health standards to address future AMR challenges.

**Figure 1.**
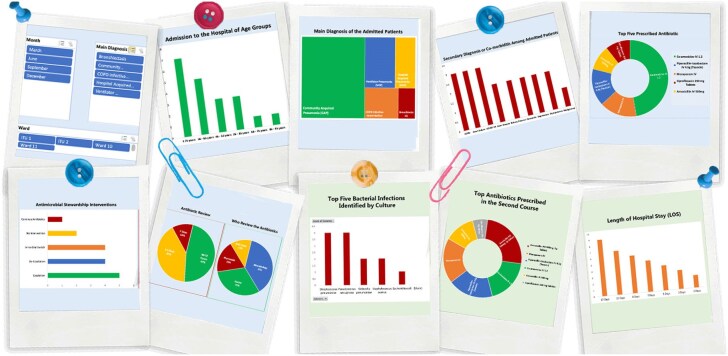
Antimicrobial stewardship (AMS) Dashboard: trends in antibiotic use, infections, and stewardship outcomes.
